# myGRN: a database and visualisation system for the storage and analysis of developmental genetic regulatory networks

**DOI:** 10.1186/1471-213X-9-33

**Published:** 2009-06-06

**Authors:** Jamil Bacha, James S Brodie, Matthew W Loose

**Affiliations:** 1 Institute of Genetics, University of Nottingham, Nottingham, UK

## Abstract

**Background:**

Biological processes are regulated by complex interactions between transcription factors and signalling molecules, collectively described as Genetic Regulatory Networks (GRNs). The characterisation of these networks to reveal regulatory mechanisms is a long-term goal of many laboratories. However compiling, visualising and interacting with such networks is non-trivial. Current tools and databases typically focus on GRNs within simple, single celled organisms. However, data is available within the literature describing regulatory interactions in multi-cellular organisms, although not in any systematic form. This is particularly true within the field of developmental biology, where regulatory interactions should also be tagged with information about the time and anatomical location of development in which they occur.

**Description:**

We have developed myGRN (), a web application for storing and interrogating interaction data, with an emphasis on developmental processes. Users can submit interaction and gene expression data, either curated from published sources or derived from their own unpublished data. All interactions associated with publications are publicly visible, and unpublished interactions can only be shared between collaborating labs prior to publication. Users can group interactions into discrete networks based on specific biological processes. Various filters allow dynamic production of network diagrams based on a range of information including tissue location, developmental stage or basic topology. Individual networks can be viewed using myGRV, a tool focused on displaying developmental networks, or exported in a range of formats compatible with third party tools. Networks can also be analysed for the presence of common network motifs. We demonstrate the capabilities of myGRN using a network of zebrafish interactions integrated with expression data from the zebrafish database, ZFIN.

**Conclusion:**

Here we are launching myGRN as a community-based repository for interaction networks, with a specific focus on developmental networks. We plan to extend its functionality, as well as use it to study networks involved in embryonic development in the future.

## Background

Interactions between genes and their products form complex cascades that can regulate biological processes. Collectively, these interactions are commonly referred to as genetic regulatory networks (GRNs), the elucidation of which is key to our understanding of the mechanisms underlying biological processes [1]. For example, knowledge of a GRN for a biological process facilitates systematic prediction of the consequences of changes within it [2]. Similarly, comparing topologies of networks between different species will inform our understanding of evolution [3].

The ability to compile GRNs in single celled organisms has expanded dramatically in the last few years [4-6]. Visualisation of these networks is relatively straightforward as all the interactions occur within a single cell. Multi-cellular organisms pose a more complex problem; effectively they consist of multiple networks within individual cells that interact with one another. Here we present a database system, myGRN, which enables users to construct, visualise and analyse GRNs in multi-cellular organisms. While our approach can be used for GRNs in any context, it has particular advantages for GRNs in developmental processes.

### Network Construction

There are two main approaches currently in use to construct networks [7]. The first is by direct experimentation, with interactions systematically tested and verified in the laboratory. The mapping of interaction networks can often be a long-term focus of a laboratory, or even multiple laboratories [8]. With the development of high-throughput methods, the availability of sequenced genomes and bioinformatics methods, significant sections of a regulatory network can be elucidated as a result of a single study [9,10]. Similarly, tools have been developed for inferring networks from expression microarray experiments and predicted transcription factor binding sites [11-13].

The second method is to exploit information already in the scientific literature on genetic and molecular interactions in a wide range of species. However, finding, collating and integrating this data is laborious and time consuming. Building such networks requires extracting the essential experimental data from multiple papers and assessing its rigor and validity. Using the conventional approach of simple text searches using PubMed or similar services can be an inefficient process, as searches often return hundreds of results per pair of putative interacting genes. Such a large result set is laborious to comprehensively review, and relevant papers may be missed.

To automate this process, a number of open source [14,15] and proprietary [16,17] tools have been developed that use natural language processing (NLP) algorithms to search online databases and extract interaction data from abstract text. As aids to manual curation, these tools are useful, but currently have high false positive rates. Despite these challenges, a number of groups have constructed and published detailed regulatory networks based on exhaustive manual and automated literature surveys, often alongside direct experimentation [18-23].

A number of molecular interaction databases that are backed by dedicated curation teams have been developed [24-31]. As well as a continuously updated reference source, it is possible to submit high-throughput interaction data to one of these databases alongside publication [25]. Many focus on a specific type of interaction (e.g. MINT[26], DIP[24]), or results from particular sets of experiments or species (e.g. Fly-DPI[27]), while some act as repositories for molecular interactions including both protein-protein and protein-DNA (e.g. BioGRID[28], BIND[29], IntAct[30]). Other services aim to integrate the data from multiple interaction databases to produce a single searchable resource (e.g. BNDB [31]). However, none of these focus explicitly on interactions within GRNs, instead containing mainly protein-protein interactions.

At the rate at which new interactions are being identified, even dedicated curation teams cannot ensure complete coverage of the data. This can be addressed, in part, by community-based curation. This model is already being applied successfully, for example in annotating *cis*-regulatory elements and polymorphisms that affect gene expression [32] and the curation of interaction data (e.g. CBioC [33]).

### Network Visualisation

Alongside the problem of network data acquisition and curation, exploring interaction data and networks is non-trivial. Most current databases return lists of interactions in response to a search query. The degree to which these data can be searched varies widely, from multi-parameter searching (IntAct [30]), to searching for a single interacting molecule and all its interactions (such as in MINT [26]). These approaches are limited, as interaction lists do not intuitively describe network topology. Stand-alone tools focused on displaying network diagrams are available which address this problem. Particularly notable are Cytoscape [34] and BioTapestry [35,36]. Cytoscape is a general network analysis platform that focuses on the analysis of network topology, and is amenable to third party development of plugins. BioTapestry, is designed to represent developmental processes [35,36], and includes automatic layout features and the ability to associate tables of experimental data with specific interactions. The resulting network models can be exported to SBML (systems biology markup language) but network construction and editing is non-trivial. For example, BioTapestry networks are not dynamically generated, but have to be manually assembled. Recently, a number of visualisation tools such as Osprey [37], VisANT [38] and BioNetBuilder [39], have been released which are able to draw network diagrams directly from database searches. However, these applications lack the rich layout of BioTapestry, as they do not include information on the location of interactions in time or tissue, a feature particularly important when investigating developmental networks.

The success of these approaches depends on the underlying data. The majority of interaction databases do not have community-based models of curation, so inclusion of novel interactions by users is difficult. Likewise, most databases focus on networks within single celled organisms, largely a consequence of the volume of data available compared to multicellular eukaryotes. Furthermore, developmental processes in eukaryotes involve intercellular signaling between cells in different tissues. Therefore, the inputs and outputs from a given gene can differ significantly between cells or tissue types. Conceptually, multicellular organisms represent a series of interconnected networks, each within individual nuclei. To integrate these multiple networks into an overall organism view, Davidson has proposed two alternative methods; the 'view from the genome' and the 'view from the nucleus' [8]. In the former, all inputs and outputs for a given gene are integrated, revealing the overall network architecture at each gene's promoter. In the latter, the individual inputs and outputs for a given gene active in specific nuclei are described. BioTapestry displays networks as a 'view from the genome' overview, with subsidiary displays providing a 'view from the nucleus' as defined by the user.

### A database driven solution

As interaction data becomes available for multi-cellular organisms, it is possible to generate genome-centered views of the GRNs for developmental processes [1,8,19-22]. However, to best represent developmental networks, it is necessary to include an appropriate display of timing and location information about specific interactions (the view from the nucleus). BioTapestry addresses this problem by allowing the user to specify different views from the nucleus distinct from the overall view from the genome [36].

As an alternative approach, we present a computational platform called myGRN (my Gene Regulatory Network) that allows individuals to work with network data. Importantly, genes and interactions can be tagged with expression location and timing information, allowing different 'views from the nucleus' to be dynamically generated from the database. We have also developed a network visualiser, myGRV (my Gene Regulatory Viewer). A network parser that allows querying of specific subsets of a network integrates these two components. The network parser also provides the same functionality to other visualisation tools including BioTapestry by exporting networks in a variety of formats. Our goals were to develop a system that can:

1) Act as a repository for interaction data (both direct experimental evidence and literature mining).

2) Integrate data from multiple labs to generate unified networks.

3) Provide a simple interface that allows for complex and flexible querying of the data.

4) Dynamically construct networks from an underlying dataset and provide these for export to other applications.

5) Produce visualisations that are relevant to exploring developmental regulatory networks.

## Construction and content

### myGRN: Database and Web Interface

**Figure 1:**
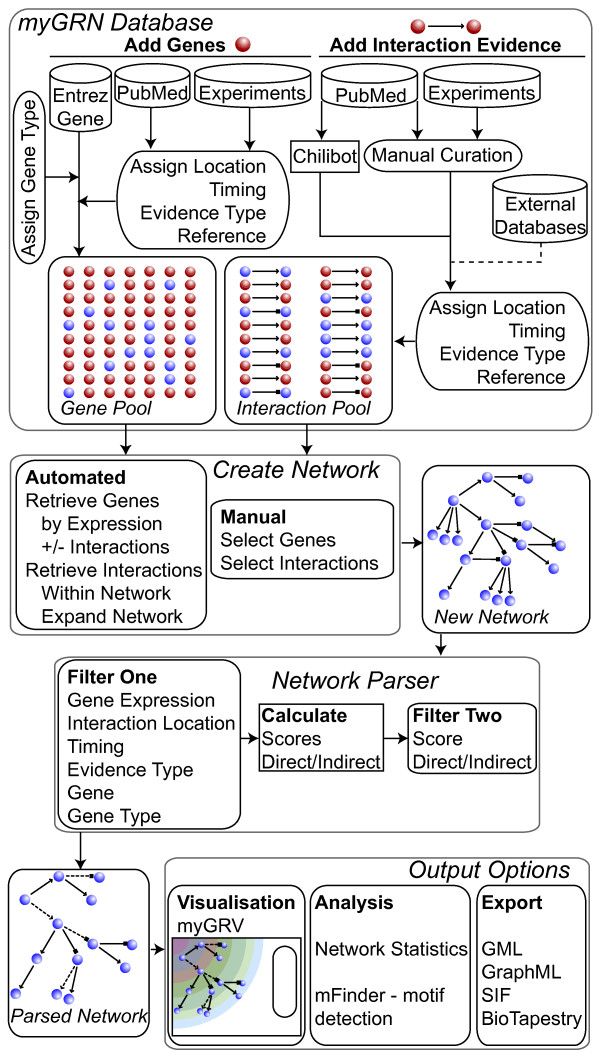
**Structure and utility of myGRN**. The myGRN database is composed of genes and the interactions between them. Genes themselves are stored with Entrez IDs, and users can add expression data tagged with experimental metadata. Interactions are directed links between the genes in the database, and are tagged with metadata detailing the experiments and publications supporting the interaction. Networks are custom groups of genes and interactions generated by users, either by adding interactions individually or using the populate tools. Once a network is assembled, it can be analysed using the network parser, which applies a series of filters to generate a subset of the network. This can then be visualised directly in myGRV, analysed for network motifs in mFinder, or exported in a range of exchange formats.

myGRN is a relational database and website built in MySQL/PHP, available at . A flow diagram illustrating the relationships between, and use of, the database, website and subsequent visualisation, export and analysis functions is shown in Figure 1. The database is organised around genetic regulatory interactions, which are stored as directed links between pairs of interacting genes. Individual genes are unambiguously identified by Entrez ID and tagged with basic gene function information. myGRN makes an initial inference of gene function based on Gene Ontology information, which can be subsequently edited by users.

Each interaction between gene pairs is stored non-redundantly, but can be tagged with multiple sets of metadata. This includes the nature of the interaction (activates, represses or unknown), the experimental evidence validating the interaction and the associated references. Importantly for the representation of developmental networks, the metadata also includes the physical location of an interaction within the organism and developmental stage at which it occurs. Likewise, genes are also associated with expression data, including anatomical location, timing, evidence and associated references.

### Metadata

Individual users specify metadata for a given interaction. To maximise data integrity and allow systematic interrogation of the database, pre-defined ontologies have been included for various metadata types. myGRN supports two methods of specifying location; anatomical location or cell type. Anatomical locations are obtained from anatomy ontologies specific to each of the 10 multicellular species currently supported by myGRN; *C. elegans*, *C. intestinalis*, *D. rerio*, *D. melanogaster*, *G. gallus*, *H. sapiens*, *M. musculus*, *R. norvegicus*, *X. laevis *and *X. tropicalis*. These are publicly available from the OBO Foundry [40], except *G. gallus*, for which the ontology was adapted from GEISHA, a chicken *in situ *hybridisation database [41]. Cell types are specified according to the cell type ontology, also available at the OBO Foundry [42]. To accommodate interactions verified in cell-lines, we have added the 1084 listed cell lines in the European Collection of Cell Cultures (ECACC). Developmental time points for interaction and expression data are specified according to accepted time series for each species. myGRN also supports data entry for two yeast species, *S. cerevisiae *and *S. pombe*. These have no anatomy ontology, and so are only annotated with timing according to the cell cycle.

We include a set of 31 evidence types that users can associate with an interaction or set of expression evidence, alongside a free text comment detailing how the evidence supports the interaction (Table 1). Classifying the experimental evidence in this way facilitates subsequent manipulation of the data as discussed below.

**Table 1:** Interaction evidence types supported by myGRN

**Evidence Type**	**Evidence Description**	**Score**
Mined from Literature	Inference of an interaction from *in silico *textual analysis of a published paper by a natural language processing (NLP) algorithm.	0

***Binding site/Promoter Interaction:***		

Transient transfection	Perturbation of expression of a reporter under control of the downstream gene promoter on disruption of upstream gene expression	10

Stable transgenic line	Perturbation of expression of a reporter under control of the downstream gene promoter on disruption of upstream gene expression	10

Protein synthesis inhibition	Demonstration of a direct interaction by inhibition of intermediate protein production using cyclohexamide or other inhibitors.	50

*In silico *prediction	Prediction of a binding site for the upstream gene product in the downstream gene promoter using in silico methods.	2

Elecrophoretic Mobility Shift Assay (EMSA)	EMSA showing direct binding between the upstream gene product and a fragment of the downstream gene promoter in vitro.	25

DNase Footprinting	Protection of a putative binding site in radiolabelled DNA containing the downstream gene promoter by the upstream gene product.	25

ChIP-on-chip	Chromatin Immunoprecipitation using antibody for the upstream gene, and analysis of the binding site locations on a microarray.	25

***Expression Evidence:***		

Stable transgenic reporter	Transgenic organism carrying a reporter gene under the control of the promoter of the gene of interest.	25

Transient transgenic reporter	Transient transfection of a construct carrying a reporter gene under the control of the promoter of the gene of interest.	25

SAGE	Detection of a sequence tag from an mRNA in a Serial Analysis of Gene Expression experiment.	10

RT-PCR	Measurement of mRNA by Reverse-Transcription Polymerase Chain Reaction.	10

Primer extension	Extension of a gene-specific primer using an mRNA template.	10

Nuclease protection	Measurement of mRNA by hybridization with an antisense probe followed by ribonuclease digestion of unbound RNA.	10

Northern Blot	Measurement of mRNA by northern blot.	10

*In situ *hybridisation	Measurement of mRNA by in-situ hybridization.	25

cDNA Library	Isolation of a gene from a cDNA library.	10

Array	Measurement of mRNA on an expression DNA microarray.	10

Western blot	Measurement of protein by western blot.	10

Mass Spectrometry	Detection of protein from cell or tissue extract using mass spectrometry.	10

Immunohistochemistry/immunocytochemistry	Measurement of protein by antibody staining in tissue slices or cultured cells.	25

***Perturbation Evidence:***		

Mutation of binding site	Site-directed mutagenesis of a known binding site of the upstream gene in the downstream gene's promoter leads to a decrease in activity of a reporter gene	25

*In silico *inference	Inference of an interaction from mathematical modelling of experimental data such as microarray time course.	2

Transient transgenic	Transient transfection of a DNA construct containing the upstream gene, controlled by a constitutive or inducible promoter.	10

Stable transgenic	Stable transgenic cell or organism line containing the upstream gene controlled by a constitutive or inducible promoter.	10

RNA-based overexpression	Introduction of synthetic or purified mRNA of upstream gene into target cells or tissues.	10

Protein-based overexpression	Introduction of synthetic or purified protein product of upstream gene to target cells or tissues.	10

Transgenic knock-out	Stable transgenic cell or organism line with the upstream gene knocked-out either functionally or by expression.	25

RNAi	RNA interference-based knock-down or silencing of the upstream gene.	25

Morpholino	Morpholino-based inhibition of translation of the upstream gene mRNA.	25

Molecular Inhibitor	Use of an inhibitor molecule to reduce or eliminate the function of the upstream gene product.	10

Dominant-negative protein expression	Insertion of a dominant-negative version of an upstream regulator into a system reduces or eliminates expression of the target gene.	10

There are 31 evidence types divided into three categories. Binding site evidence demonstrates a molecular interaction between the upstream gene product and the promoter of the downstream gene. Expression evidence shows that the interacting genes are co-expressed (activators) or inversely expressed (repressors) in a given tissue. Perturbation evidence shows that changes to the expression of the upstream gene lead to perturbed expression of the downstream gene. We have given each experimental evidence type one of four possible scores (50 – conclusive, 25 – strongly indicative, 10 – indicative or 2 – suggestive) reflecting how well it supports the appropriate category. Interaction evidence that is extracted from the literature with an NLP algorithm is not placed into any of these classes and does not contribute to the scoring of an interaction.

### What is a network?

Users can group any collection of genes and interactions into a network within myGRN. For example, a user can create custom groups of interactions that govern a biological process, or are all inferred from a single experiment. Individual interactions can be members of any number of networks, and users can add any referenced interaction already in the database to their own networks. Each network is associated with metadata including comments by the creators, published references on which the network is based and whether the network is publicly available or private.

All interaction data and networks associated with a publication in myGRN are freely available. Any user can contribute to myGRN by adding experimental evidence of interactions or gene expression linked to a published paper. Users can create private networks that are accessible to members of their own lab, allowing them to integrate unpublished data within the published body of knowledge. Users can share private networks with collaborating groups, whilst withholding the network from full public access. Notably, any data curated into the database and associated with a publication is potentially visible to all, even though it may be within a private network. Other users can include these interactions in their own networks, encouraging open access data sharing. To facilitate this, we provide a sophisticated 'populate' tool, discussed further below, which allows users to add genes to a network based on their expression and then scavenge interactions between these genes from the wider database.

### The myGRN Network Parser

The network parser allows users to carry out advanced searches of the database, or create sub-networks for subsequent analysis, visualisation or export. The parser can filter the metadata and/or select a specific set of genes to generate sub-networks. Optionally, the user can include the neighbours of the selected genes to expand the set. The depth can be set up to three layers from the selected genes. A final filter can be applied based on a calculated interaction score and the user can choose to exclude interactions likely to be indirect. These scores are calculated based on the data that passes all other pre-applied filters and are discussed further below.

The data extracted by the parser can either be passed directly to another tool in the myGRN system, or exported for analysis with third party applications. The current tools available within myGRN are the myGRV visualisation tool and mFinder, a third-party motif detection tool [43].

### Assessing and scoring interactions

By design, single interactions can be supported by multiple evidence entries in the database. In these cases, myGRN amalgamates the metadata to display a single interaction where the locations, timings and evidence reflect the total information available. The experimental evidence types in myGRN are classified into three categories according to which of the following statements they support (Table 1) [8]:

1. Binding site/Promoter Interaction: The upstream gene product binds a regulatory element of the downstream gene.

2. Expression Evidence: The two genes are expressed consistent with the proposed interaction between them.

3. Perturbation Evidence: Perturbation of the expression of the upstream gene alters the expression of the downstream gene.

If an interaction is supported by evidence in each of these three categories, myGRN designates it as a direct interaction (i.e. the upstream gene product directly binds to a regulatory element of the downstream gene and affects its transcription). Interactions that do not fulfill this criterion are classed as indirect (i.e. it is not possible to exclude the presence of an intermediate gene). The only exceptions to this rule are interactions supported by protein-synthesis inhibition experiments and interactions imported from third-party NLP applications. Protein-synthesis inhibition experiments demonstrate interactions to be direct in the absence of other evidence. NLP derived interactions are not assigned to a specific category, as they are not currently tagged with the relevant metadata.

After allocation as either direct or indirect, myGRN generates a score reflecting the degree of support for an interaction. This is not a measure of activation or repression, but instead allows users to assess the quantity and quality of evidence available for a given interaction. Each evidence type has been assigned a score reflecting how well it supports an interaction (Table 1). The combined score in each evidence category is calculated, with the maximum possible score for any one category capped at 50. We take this cap, although arbitrary, as indicating conclusive support for that category. The three category scores are summed and this result is presented as a percentage total score.

The total score for an interaction is dynamically calculated from the evidence in the database. Thus the scores update when new evidence is added, and they respond to filters applied using the network parser. For example, if a user excludes a particular class of evidence, the scores produced by myGRN are lower than if the filters were not applied. NLP derived interactions are not currently assigned a score, as the evidence type used to infer the interaction cannot be determined automatically.

### myGRV: Interactive Network Viewer

myGRV is an interactive visualisation tool, written in Adobe Flash, for viewing and analysing networks built from data in myGRN. It is designed to be web-based and platform-independent, requiring only the freely available Adobe Flash plug-in. Its input is an XML based file generated by the myGRN network parser. This file contains complete information on all the genes and interactions in the dataset produced by the parser. myGRV interprets the XML file and generates interactive network diagrams from which the evidence for each interaction can be explored.

### mFinder Implementation

An important emerging tool for understanding the function of networks is the identification of network motifs, small groups of genes that form sub-networks within a larger network. These motifs are thought to carry out information-processing functions within a network, the behaviours of which have been reviewed elsewhere [44]. Identifying motifs can contribute to our understanding of the mechanistic role of individual components within a network. We have incorporated mFinder, a third party network motif detection algorithm developed by *Kashtan et. al*. [43], into myGRN. mFinder identifies network motifs of 3 to 6 nodes within a given network graph. The mFinder source code is available from the original authors for compiling and running as a stand-alone program. To facilitate its use with myGRN, a network generated by the parser can be directly piped into mFinder. Within myGRN, motifs detected by mFinder can be viewed graphically. Users select a motif type and a gene of interest, and the applet generates an interactive plot of that gene, all neighbouring genes involved in that motif, and all the interactions between them. Our online implementation of mFinder restricts users to searching for motifs of 5 or less nodes and limits the number of iterations that can be carried out for statistical analysis to 2000. To access the full range of mFinder parameters, we provide a downloadable Perl script. This script allows mFinder to use GML files exported from myGRN as input. It reformats the mFinder results to show the original gene names and the nature of the interaction (activation or repression) as well as allowing the user to directly determine the motif structure, features not available with the original mFinder.

### Chilibot implementation

To aid manual curation we have incorporated access to Chilibot, a third party NLP algorithm developed by Chen and Sharp, into myGRN [14]. This identifies candidate molecular interactions by analysing PubMed abstracts. myGRN interfaces with the Chilibot server via an API, and can submit queries and retrieve results directly from a user's network. myGRN parses the results of Chilibot queries, and stores them in the database to avoid the need for future remote access. Users can mark results of interest and add them to a curation queue. When users choose to curate these results as interactions, the information on the interaction partners and the paper are passed to the same set of pages used to curate interactions *de novo*. Users then simply fill in the missing information.

### Content

**Figure 2:**
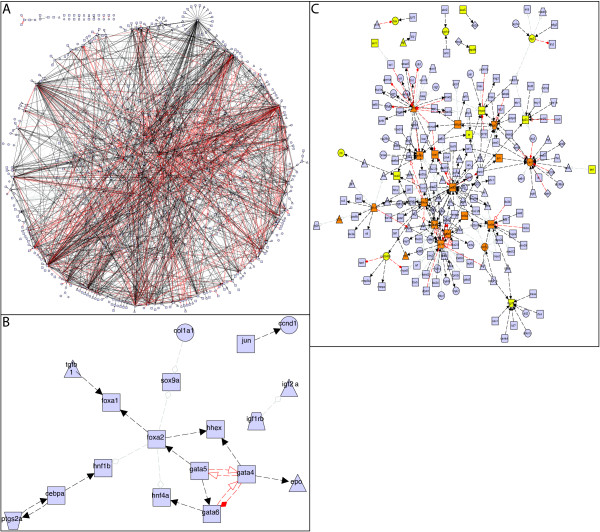
**Zebrafish development networks according to gene expression**. (**A**) The complete set of Zebrafish interactions currently stored within myGRN. (**B**) A subset of genes from A that are expressed in the liver, and the interactions between them. (**C**) Expansion of B to include all interaction partners, regardless of expression location. Orange coloured genes are present in (B), yellow genes are expressed in liver but do not interact with other liver expressed genes, blue genes are not expressed in liver. These networks were exported from myGRN as graphML and visualised using the yEd Graph Editor . Different node shapes represent gene types: triangle = signal (ligand), rectangle = transcription factor, trapezoid = receptor, circle = terminal marker. Line styles represent interaction types: solid=direct, dashed = indirect, black arrow = activation, red arrow = repression, grey diamond = unknown.

In the initial release of myGRN, we have populated the database with a network of interactions involved in zebrafish development, compiled by Chan *et. al*. using the MedScan NLP algorithm to extract interactions from published papers [23]. We received the full set of interactions, involving 852 genes, directly from the authors. Of these, we were able to determine the Entrez IDs of 832 genes. We then removed any interactions for which one of the genes did not have an Entrez ID, or for which we could not locate the reference in PubMed, leaving a set of 2385 interactions (the full network can be seen in Figure 2A). We have run our mFinder implementation on this zebrafish network, and have made the statistics [see Additional file 1] and motif list [see Additional file 2] files freely available. Alongside this, we have included expression data for all zebrafish genes obtained from the ZFIN database [45]. We excluded ZFIN expression data that was not associated with a publication, where the developmental stage was not defined, or where the gene was not associated with an Entrez ID. Our final expression dataset contains 20054 expression entries for 3044 genes, including the majority of those in the *Chan et. al*. dataset. Importantly, this allows users to select, visualise and export subsections of the zebrafish network based on gene expression patterns. We also plan to include and expand our previously published GRNs for haematopoiesis in mouse [21] and mesendoderm development in *Xenopus *[22].

## Utility and discussion

The interface to myGRN has been built to allow any user to easily browse networks, enter data and view networks without specialist skills.

### Data exploration

There are two methods available to explore interaction data within myGRN. Firstly, users can search for a gene by name and view a list of its interactions. Secondly, users can select a specific network and browse the list of genes within it. Selecting any gene will reveal its upstream and downstream connections in that network. Selecting any of the connecting genes will re-centre the view on that gene and reveal its targets and regulators and so on. All the metadata associated with an interaction is available in this view. The cumulative evidence for each interaction is assessed by the myGRN scoring system providing the percentage score and direct/indirect designations.

### Adding data to myGRN

Any registered user can add data to myGRN and construct their own networks. Registration allows users to manage the sharing of unpublished data with others and reduces the possibility of malicious data submissions. The myGRN website includes forms for the addition of interactions and expression data. Users can specify all the metadata associated with each entry (see Construction and Content). The forms interface directly with NCBI Entrez, allowing the direct search and import of genes from Entrez Gene and papers from PubMed.

As well as submitting new interactions, users are able to search for existing interactions from other networks, and import these into their network. While browsing a network, myGRN will highlight where a gene has other interactions of potential interest, and those can be added individually. myGRN also includes a set of importing tools for populating a network with existing data. Genes can be added according to their expression in one or more tissues and their involvement in interactions.

Interactions can then be imported from the database between all the genes in the selected set. In addition, one or more genes from the network can be submitted as a search. In this case myGRN imports all interactions involving the submitted genes in the database whether or not their interaction partners are already in the network. In this way, networks can be grown from individual collections of genes. Interactions not associated with a publication cannot be imported this way.

These tools are a powerful way of interrogating interaction and expression datasets to produce a putative network for a biological system. We have demonstrated this by integrating the zebrafish interaction network mined from the literature by Chan *et. al*., with the expression data from ZFIN (Figure 2A). Such large networks can be reduced by including only those genes expressed in a specific subset of tissues, for example, the liver (Figure 2B). In this case, myGRN identifies 33 genes, 18 of which have 21 distinct interactions between them. The network can then be expanded by searching using a subset of the genes and importing all further interactions involving at least one of them, regardless of the expression of its interacting partner (Figure 2C). This identifies a further 176 genes and 288 interactions of potential interest. As the data within myGRN grows, these tools will allow users to rapidly develop and analyse networks.

### Curating interactions from the literature with Chilibot

To aid retrieval of interactions from the literature, myGRN uses Chilibot to identify papers containing potential interactions between genes of interest [14]. Users can select up to 5 genes of interest from any given network for submission to Chilibot. Chilibot searches PubMed abstracts for sentences that imply an interaction between any two of the submitted gene set. The results are returned and stored in myGRN, allowing users to review the papers, marking results as informative or non-informative (in which case they are discarded). Informative results are placed in a curation queue and interactions can be entered into myGRN, complete with evidence and other metadata.

### Data sharing

One of the distinguishing features of myGRN is that users can construct networks with unpublished data alongside interactions from the published literature. In this sense, it serves as a tool and a data repository for labs to store and analyse their nascent GRNs. However, this raises confidentiality concerns for users who may not wish to share results prior to publication and unpublished results not subject to peer review cannot be verified. To address these issues, all users require an account enabling them to submit data, and this account is affiliated with one or more labs. This information is used by myGRN to manage data sharing within the database. Unpublished interactions submitted by a user can be seen by any other user who is affiliated to the same or collaborating laboratories, but are not available to any other user.

Users can also designate an entire network as private. This network will then only be available to other registered users in the same lab. However, any interactions entered into a private network and associated with a publication are available to all users. In this way, published data is never hidden from any user, but networks designated as private are not visible to third parties to browse. For users with further concerns about data confidentiality, the database schema, website code and public data are freely available for academic users to use locally.

### Network visualisation, analysis and export

As well as the static web-based view of a network, users can generate sophisticated network diagrams and exportable datasets, and run analyses using the myGRN parser. myGRV plots interactive network diagrams, or subsets thereof, that allow users to explore network topology. myGRV is optimised to view smaller networks and subsets of larger networks. Once the network includes more than 50 nodes, it is difficult to explore visually at the level of detail offered by myGRV and we suggest exporting networks from myGRN for analysis in other networks viewers (see below for details). Within myGRV, networks are displayed using one of three methods, hierarchy, force-spring or spatio-temporal.

**Figure 3:**
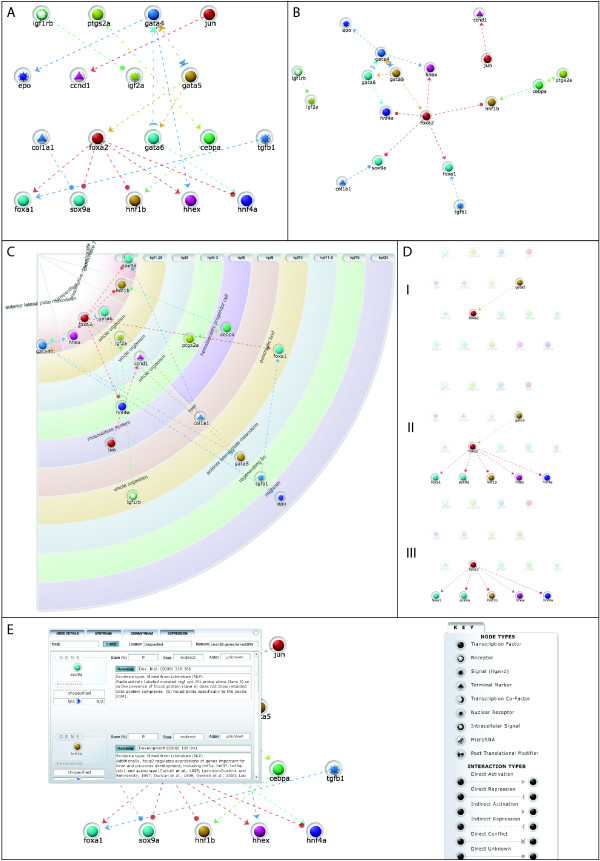
**Layout methods in myGRV**. The same network as Figure 2B, but viewed using myGRV. (**A**) shows a hierarchical layout, (**B**) shows a force-spring layout and (**C**) shows the layout in spatio-temporal view. Here genes are positioned according to the tissues in which they are expressed. (**D**) shows the same network as (A). In this case FoxA2 has been selected and (**Di**) highlights upstream interactors, (**Dii**) highlights genes immediately connected to the selected gene and (**Diii**) only highlights downstream targets. (**E**) shows an example of browsing the network detail via the myGRV interface. The key shows how different gene types and interactions are represented within myGRV.

*Hierarchy*: this layout positions genes on the page according to their inputs and outputs (Figure 3A). Genes without inputs are placed at the top of the page with their targets below. Genes without outputs tend to the bottom of the page. *Force-spring*: This treats each gene as an object within a physical environment that is attracted to connected genes, modelled as a spring between two genes (Figure 3B). All genes repel each other simultaneously as electrically charged particles. This layout works well for networks with less than 50 nodes, but is computationally intensive for larger networks. To display very large networks, better results will be obtained by using the network parser to export the network to a third-party visualiser. *Spatio-temporal*: We developed this layout specifically to display developmental networks (Figure 3C). Developmental time flows diagonally across the screen and genes are positioned by time, either according to gene expression or interaction timing data. Within each time stage, genes are grouped by the anatomical location in which the interaction is occurring. Each gene is shown once on the network diagram. Where a gene is expressed or interacts in multiple tissues, it is displayed in the tissue where the earliest of these occurs. Thus users can generate networks dynamically from the database and display them incorporating developmental information.

In any view, users can highlight upstream, downstream or both types of connection for an individual gene (Figure 3Di–iii). Users can easily switch between network views and can also manually arrange networks. As in the database view, the supporting evidence and location/timing information for all the interactions is available to view by individual gene (Figure 3E).

**Figure 4:**
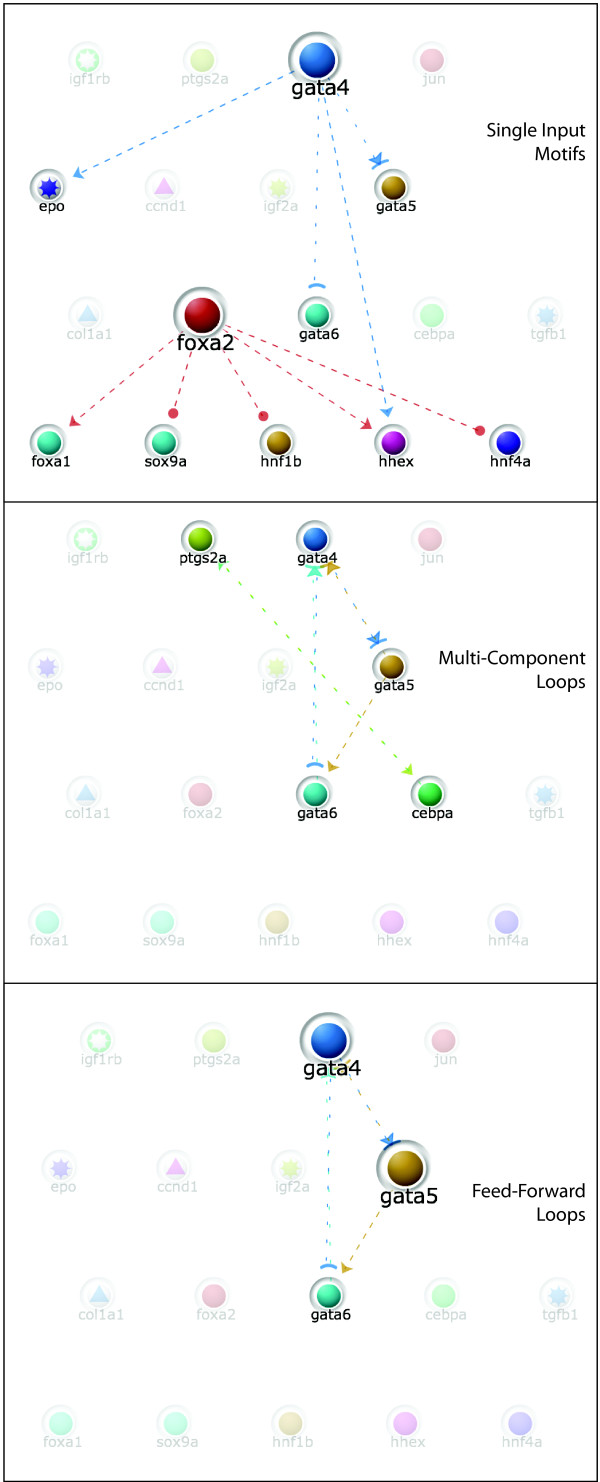
**Motif detection in myGRV**. myGRV provides basic motif detection algorithms. In this example, the same liver derived network is analysed for single-input, multi-component and feed-forward loops motifs. In the case of single-input and feed-forward loop motifs, myGRN highlights genes by altering the node size. The originating gene in the single input motifs is larger than its targets, the upstream genes in the feed-forward loop are larger than their targets.

myGRV also includes a basic motif detection tool that can search the currently displayed network for specific network motifs, including auto-regulation, feed-forward loops, multi-component loops, single and multi-input motifs (Figure 4). This is based on some of the algorithms used by mFinder, although myGRV does not run mFinder itself, and does not carry out statistical evaluation. For comprehensive motif detection, mFinder should be used directly within the website [43].

Finally, the network parser can export interaction datasets for use with other analysis programs. Currently, myGRN exports into GML, SIF, GraphML and BioTapestry CSV formats. GML is an open standard markup language for network graphs, and is limited to storing nodes and edges. Similarly, SIF (simple interaction format) files store node and edge information only. GraphML is xml-based and additionally stores metadata about the properties of the network's components. Nodes are linked to Entrez gene entries, whilst edges will link back to the myGRN database to reveal the evidence underlying a given interaction. GML and GraphML formats can be used to plot network diagrams and carry out analyses in packages such as the yEd Graph Editor. GML and SIF files are also compatible with Cytoscape [34] and other third party tools. BioTapestry CSV is a specific file format for importing data into the BioTapestry application. BioTapestry produces sophisticated network diagrams and also serves as a conduit for converting myGRN models to other common formats such as SBML. BioTapestry has an additional advantage over our current visualiser as it can display one gene in multiple locations, a feature we intend to add in the future. Combining these output formats with the advanced filters in myGRN provides a powerful set of tools for network curation and analysis.

## Conclusion

We have developed myGRN, a database and visualisation system to store regulatory interaction data and dynamically plot and analyse network diagrams. We also include tools to simplify community driven curation of interaction data. myGRN facilitates the sharing of networks and unpublished data between different groups. All the tools within myGRN are web-based and platform independent.

As a database, myGRN follows the community-based curation model, and is not intended to act as a replacement for the major interaction databases. By allowing users to submit unpublished or literature-curated data and build networks, myGRN provides a tool set for research groups to work with their data, rather than just a static repository. The ability of multiple groups to collaborate on constructing a network using their own data turns myGRN into a shared 'white board' in a way that is unique to our knowledge. The data is kept integrated within a common repository, and the networks are updated dynamically as more data is added.

Several other features distinguish myGRN from similar tools already available. The advanced filtering options provided by the network parser are more flexible than comparable databases and tools. The filtering options allow sophisticated interrogation of a network by genes, tissues, timing and/or evidence classes. The tools available to populate a network with genes by expression data provide another level of filtering, and are useful for integrating distinct interaction and expression datasets, particularly when interaction datasets may not contain sufficient metadata (such as those derived from *in vitro *experiments).

The parser results can be submitted directly to visualisation and analysis tools within myGRN, or they can be exported to a variety of file formats. This opens up the data in myGRN to a wide range of analysis tools. This is also an important distinction compared to proprietary interaction database services such as Intelligent Pathway Analysis, which do not allow the export of information-rich network representations to third party tools.

The utility of these features has been demonstrated by importing a published network of zebrafish interactions and gene expression data from ZFIN. The initial interaction set lacked information on the location and timing of interactions, and was stored in various web and spreadsheet formats that, whilst commonplace for custom datasets compiled by individuals or small groups, are difficult to interrogate and update. Their incorporation into myGRN means that the data can be updated easily, and any visualisations or analyses quickly performed. Producing network diagrams arrayed by gene expression data from this dataset becomes relatively trivial and rapid.

The myGRV visualiser supports network diagrams arranged according to the location and timing of interactions, something that is absent from most visualisation tools. This makes it ideal for studying developmental networks; users can generate individual networks for different tissues in a developmental process to compare the regulatory events taking place in each, or can quickly produce a time series of network diagrams showing how topology changes over time. Importantly, new data can be quickly and systematically incorporated into a network and immediately visualised.

The inclusion of Chilibot, an NLP tool, streamlines finding relevant papers and helps ensure more complete coverage of the literature. The implementation of a curation queue aids users to keep track of papers. To our knowledge, only one other web application, CBioC [33], links an NLP algorithm to a community curation model. However, users can only display the resulting interactions as a simple list, with no other functionality currently available.

We welcome direct submission of large datasets to myGRN and have tools in place to facilitate direct upload to our database, although currently large uploads cannot be directly performed by users. As well as providing this feature and further refining those already available, we plan to extend the functionality of myGRN. We will increase the number of export formats to provide cross-compatibility with more tools. In particular, we aim to produce exportable files that are compliant with markup languages such as SBML and BioPAX. We will also extend compatibility with BioTapestry, adding export into BioTapestry's native XML format. This will enable automatic generation of complex GRN diagrams, complete with timing and tissue information, using BioTapestry's tailored system for displaying GRNs. We will develop direct links with other databases to enable myGRN users to import data into their networks. Longer term, we plan to integrate Gene Ontology terms as metadata for genes, and allow users to automatically scavenge the database and build networks based on GO terms, in a similar way to the current import tools based on expression data. Finally, one of our major goals is to develop the facility to compare two networks systematically across species, exploiting orthology information to identify conserved network kernels.

## Availability and requirements

The database and visualiser software are freely accessible at . Creating user accounts to enable data submission is free, but is required for data entry. Any modern web browser such as Mozilla Firefox, Safari or Microsoft Internet Explorer is sufficient to access the database. A Flash Player browser plugin is required to use myGRV. This is freely available to download from Adobe website.

## Abbreviations

API: Application Programming Interface; GML: Graph Modelling Language; GO: Gene Ontology; GRN: Genetic Regulatory Network; NLP: Natural Language Processing; SIF: Simple Interaction Format.

## Authors' contributions

JB co-designed and built the database, programmed the website and user interface and drafted the manuscript. JSB programmed the myGRV visualiser and assisted in designing the web interface. MWL conceived of the project, led its design and coordination, co-designed the database and drafted the manuscript. All authors read and approved the final manuscript.

## Supplementary Material

Additional file 1 **Zebrafish Development Network Statistics**. Statistical analysis of the full zebrafish network generated by mFinder integrated within myGRN. Click here for file

Additional file 2 **Zebrafish Development Network Motifs**. The raw output of mFinder as generated by myGRN. Note that gene names are given instead of reference numbers. In parentheses after each gene name are a series of characters. '_' identifies the position of the gene and shows no interaction, '-' represents no interaction, 'A' is activation, 'R' is repression, 'U' represents an unknown interaction type. The position of these codes identifies the corresponding interaction. So, the first line from the file reads 'ascl1a (_AR) pax6a (-_-) pitx3 (--_)'. In this case, ascl1a does not regulate itself; it activates pax6a and represses pitx3. Click here for file
